# Advanced rectal cancer in an 11-year-old Sudanese patient with rare metastatic site: a case report

**DOI:** 10.1093/jscr/rjaf096

**Published:** 2025-03-05

**Authors:** Nadir Ali Hilal, Ahmed Rafei, Omeralfarouk Hussein Alameen Mohammed, Noon Mohamed

**Affiliations:** Department of Colorectal Surgery, National Center for Gastrointestinal and Liver Diseases, Khartoum, Sudan; Department of Research, National Center for Gastrointestinal and Liver Diseases, Khartoum, Sudan; University of Kharoum, Khartoum, Sudan; University of Kharoum, Khartoum, Sudan

**Keywords:** pediatric colorectal cancer, cutaneous metastasis, colorectal cancer, Sudan, case report

## Abstract

Colorectal cancer (CRC) is predominantly an adult malignancy, rarely affecting children, with an incidence of less than 1% in individuals under 20 years old. Pediatric CRC typically presents with nonspecific symptoms, leading to delayed diagnosis and poorer outcomes compared to adults. Cutaneous metastases in CRC are exceedingly rare, occurring in 0.7%–5% of cases, and have not been previously reported in pediatric patients. We present an 11-year-old male with rectal adenocarcinoma who developed cutaneous metastasis after initial treatment. Misdiagnosis and delayed presentation contributed to disease progression. Treatment included neoadjuvant chemoradiotherapy, surgical resection, and adjuvant chemotherapy. Despite initial symptom resolution, the patient experienced tumor recurrence with peritoneal carcinomatosis and subsequent cutaneous metastasis, ultimately succumbing to the disease. This case highlights the challenges of diagnosing and managing pediatric CRC, emphasizing the need for heightened clinical suspicion and improved access to diagnostic tools, especially in resource-limited settings.

## Introduction

Childhood malignancies are a leading cause of disease-related mortality and treatment-associated morbidity, with their prevalence rising globally in recent decades [1]. Among these, colorectal cancer (CRC) is typically an adult-onset disease, with incidence increasing with age; over 90% of cases are diagnosed in individuals older than 50 years [2]. However, data reveal a worrying trend in younger populations between 1999 and 2020. The incidence of CRC increased by 500% in children aged 10–14, 333% in teenagers aged 15–19, and 185% in young adults aged 20–24 [3].

Cutaneous metastases are a rare manifestation of malignancies, occurring in only 0.7%–5% of patients with cancer [4]. These metastases are frequently linked to advanced, disseminated disease, and carry a poor prognosis [5]. In CRC specifically, the incidence of cutaneous metastases ranges from 2.3% to 6% [4]. Notably, no reports to date have documented cutaneous metastasis in pediatric CRC patients, underscoring the rarity of this presentation.

## Case presentation

**Figure 1:**
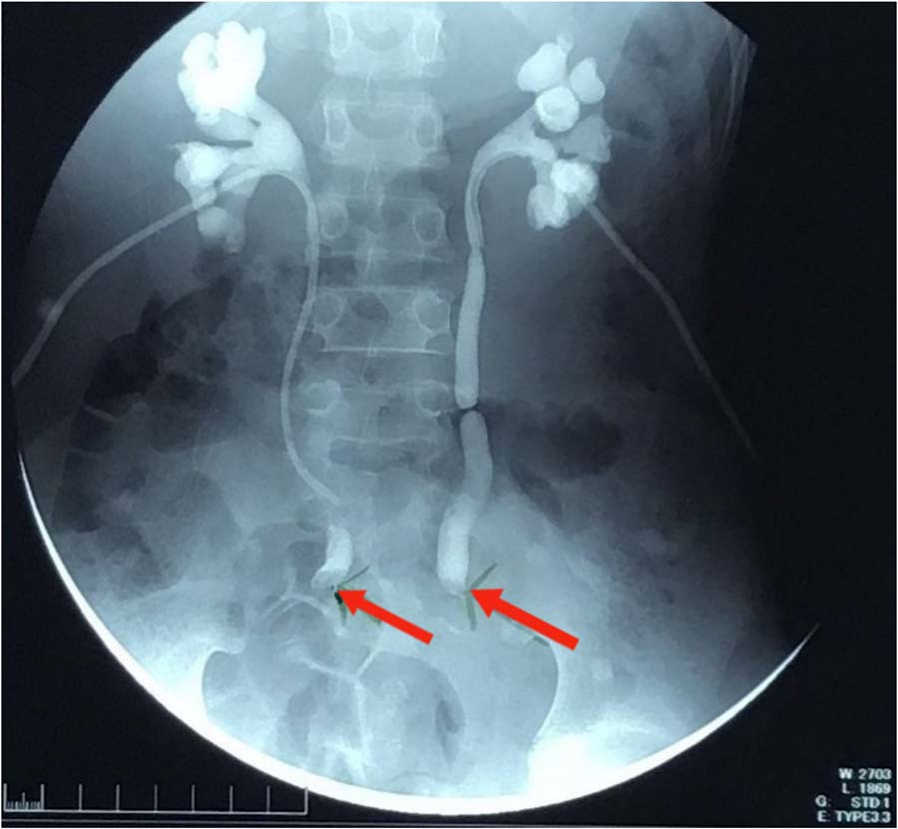
Nephrostogram showing bilateral hydroureter with complete ureteric obstruction and no dye in the bladder. The two arrows indicate complete bilateral ureterovesical obstruction.

An 11-year-old Sudanese male presented with a year-long history of intermittent lower abdominal pain, altered bowel habits, and weight loss. Four months prior to presentation, he experienced minimal, painless rectal bleeding. Later, progressive urinary symptoms culminated in oliguria. Evaluation revealed bilateral hydroureter and hydronephrosis, necessitating bilateral percutaneous nephrostomy placement after failed attempts to insert JJ stents (Fig. 1). Previously, the patient underwent an appendectomy for presumed acute appendicitis but his symptoms persisted. The family history was negative for CRC or related conditions.

On examination, the patient appeared ill, slightly pale, and underweight, with a BMI of 12. Vital signs were normal. His abdomen was soft and nontender without palpable masses, but digital rectal examination revealed a large, circumferential rectal mass located 4 cm from the anal verge. Colonoscopy could not traverse the mass, and biopsy confirmed rectal adenocarcinoma.

**Figure 2:**
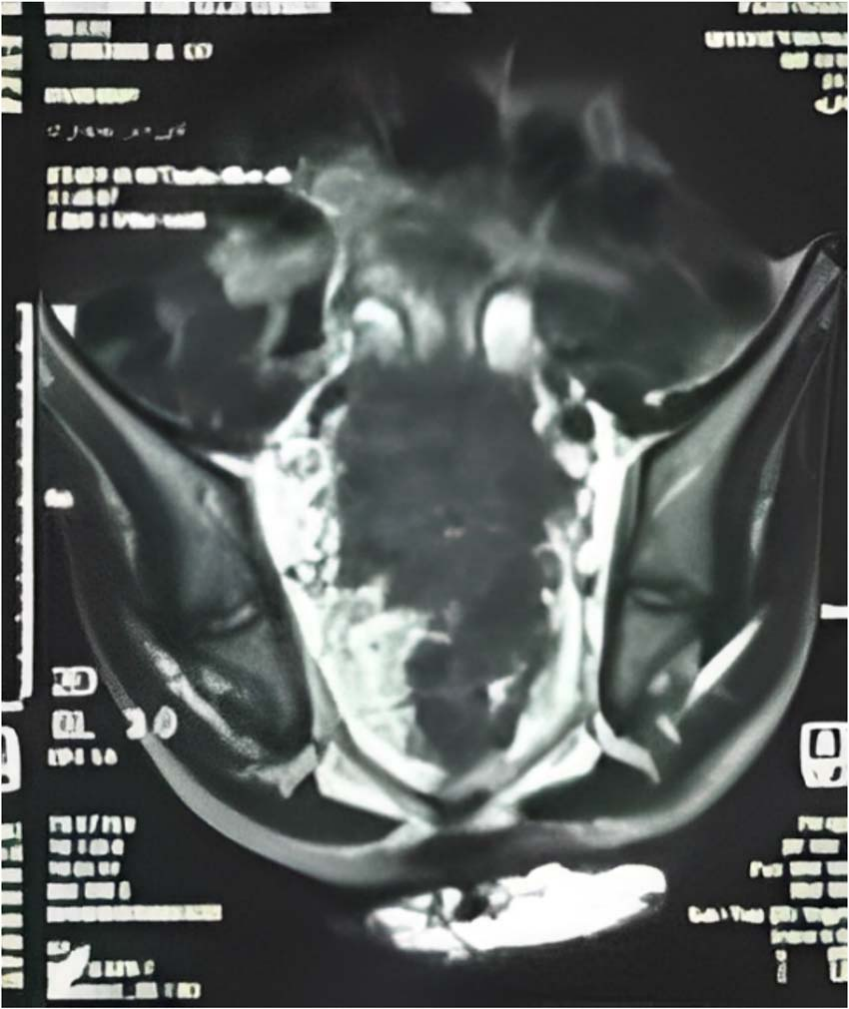
MRI sagittal view showing the tumor extending from the rectosigmoid to the upper and middle rectum, with mesorectal lymph nodes and extramural venous invasion.

Pelvic MRI (Fig. 2) identified a 5.5 × 5.5 cm rectal mass invading the posterior wall, extending to the upper rectum and rectosigmoid junction, and encasing both ureters. Enlarged mesorectal lymph nodes and extramural vascular invasion were also observed. A CT scan of the chest, abdomen, and pelvis showed no distant metastases.

The patient received total parenteral nutrition for 4 weeks, significantly improving his BMI. Multidisciplinary discussions led to neoadjuvant chemoradiotherapy comprising 5 × 5 Gy radiation and six cycles of FOLFOX chemotherapy. Follow-up pelvic MRI revealed a notable tumor response, with tumor size reduced to 5 × 2.1 cm.

**Figure 3:**
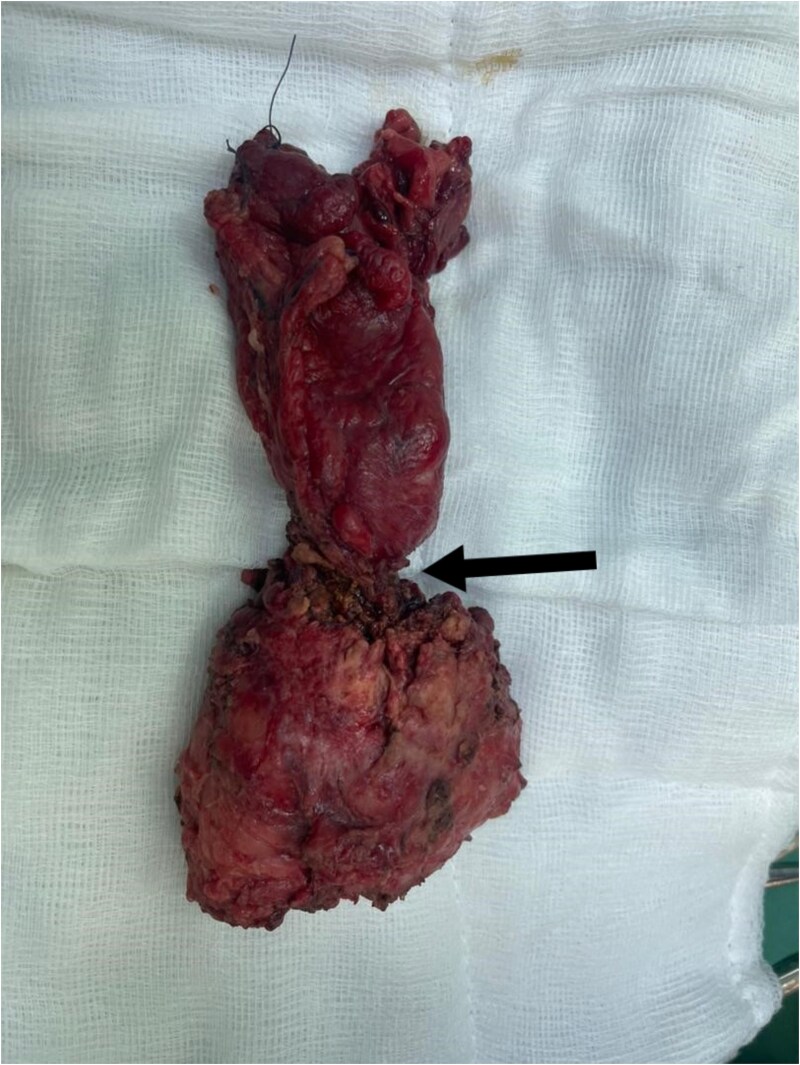
Total mesorectal excision (TME) specimen, with arrow pointing to the tumor.

Subsequently, he underwent open low anterior resection with primary colorectal anastomosis, diverting loop ileostomy, and en bloc resection of both ureters. Due to a concurrent urinary tract infection, urinary reconstruction was deferred. The right ureter remained intact, but the left ureter was involved with the tumor and required resection. The resected specimen (Fig. 3) revealed moderately differentiated adenocarcinoma with clear margins, and all six regional lymph nodes were negative for tumor infiltration (pT4a pN0 M0).

Postoperatively, complications arose when a blocked left nephrostomy tube led to urine leakage into the peritoneal cavity, causing wound dehiscence. The tube was replaced, and the infection resolved with appropriate management. The patient’s symptoms completely resolved during follow-up, and he awaited ileostomy reversal and ureteral reimplantation.

**Figure 4:**
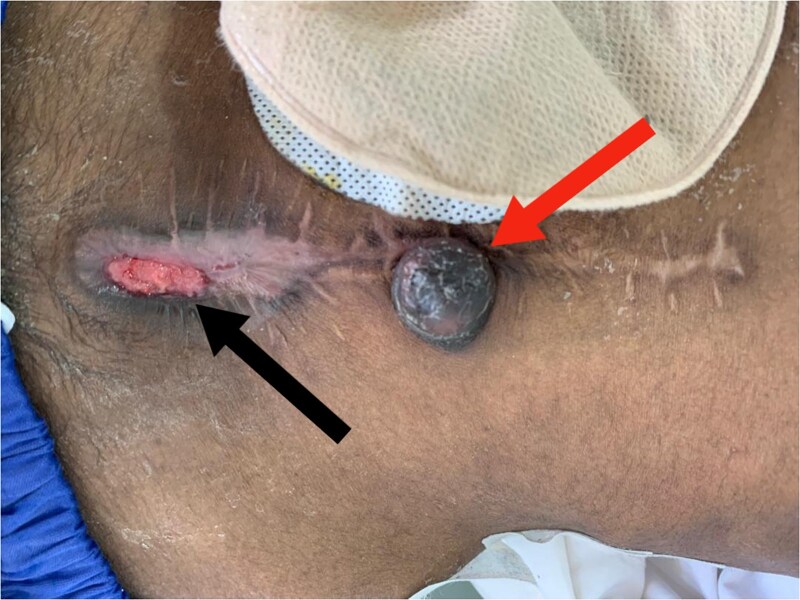
Red arrow: Sister Mary Joseph nodule. Black arrow: Wound dehiscence at the lower edge of the midline incision.

Three months later, he presented with lower abdominal pain and a rapidly enlarging umbilical swelling (Sister Mary Joseph’s nodule) (Fig. 4). A CT scan confirmed tumor recurrence and peritoneal carcinomatosis. Colonoscopy also indicated recurrent disease, and the patient was planned for adjuvant chemotherapy.

**Figure 5:**
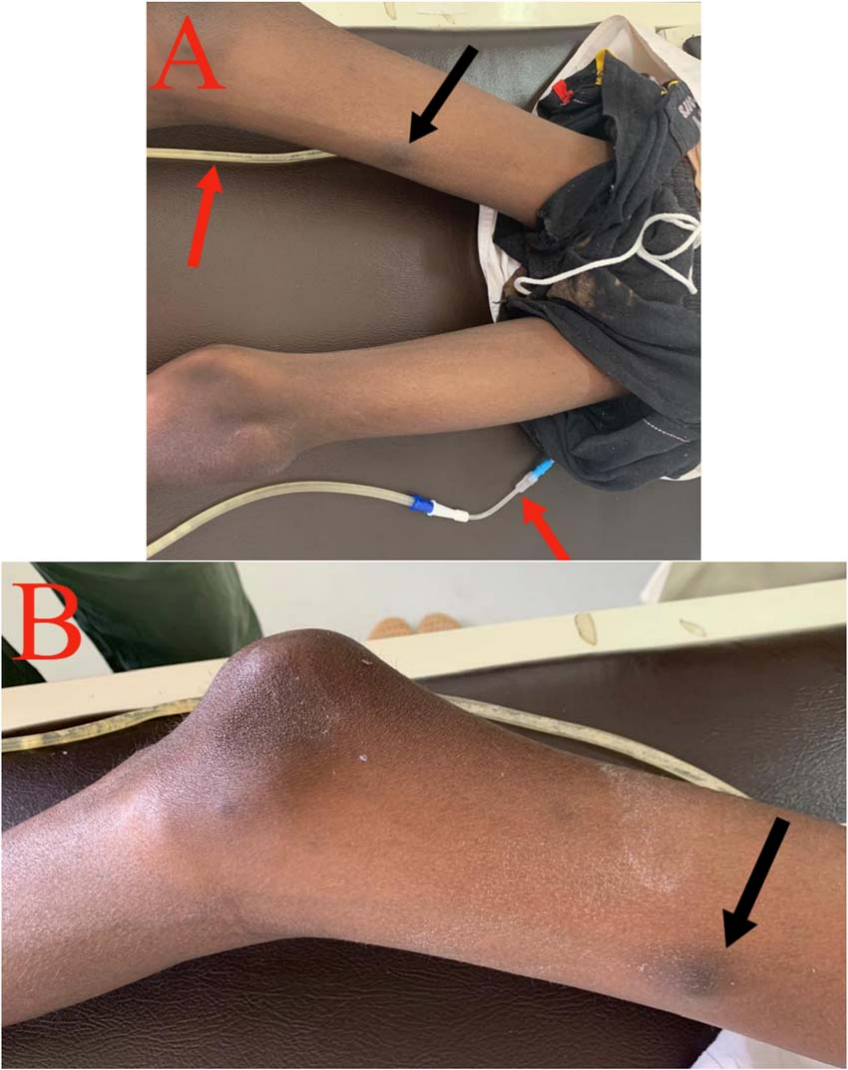
Cutaneous metastasis: (A) Black arrow: cutaneous nodule on the medial aspect of the right thigh. Red arrow: right and left nephrostomy tubes. (B) Black arrow: cutaneous nodule on the medial aspect of the right thigh.

A month later, he developed a painful 2 × 2 cm nodule on the medial aspect of his right thigh. Biopsy confirmed cutaneous metastasis (Fig. 5). Unfortunately, the patient succumbed to the disease shortly after due to extensive peritoneal progression.

## Discussion

Pediatric CRC is exceedingly rare, accounting for less than 1% of cases, with an incidence rate of approximately 1.3 per million children in the United States [6]. In India, pediatric CRC incidence is estimated at 1 in 1 000 000 malignancies annually [7]. This rarity contributes to diagnostic and treatment challenges, as most clinical knowledge is based on adult cases.

Pediatric CRC typically presents with symptoms resembling benign or infectious conditions, including abdominal pain, hematochezia, altered bowel habits, weight loss, and anemia [8]. This similarity can delay diagnosis significantly; studies report a 6-month average delay in symptomatic pediatric patients [9]. In our case, there was a 1-year delay due to initial misdiagnosis as appendicitis and limited rural healthcare access, compounded by socioeconomic factors.

Pathophysiologically, CRC follows two main genetic pathways: microsatellite instability (MSI) and APC/beta-catenin mutations. Pediatric cases often involve high microsatellite instability (MSI-H) mutations. Familial syndromes such as familial adenomatous polyposis and hereditary nonpolyposis colorectal cancer are most commonly associated, while Peutz-Jeghers syndrome and juvenile polyposis coli also elevate CRC risk [10]. Genetic screening for MSH1 and MSH2 mutations would have been invaluable in our case, but the ongoing armed conflict and limited local resources precluded this.

Histologically, pediatric CRC is more aggressive, with over 50% classified as poorly differentiated subtypes, including mucinous adenocarcinoma and signet-ring cell carcinoma. Mucinous adenocarcinoma, which accounts for 10% of adult cases, is associated with a more aggressive course and worse outcomes [11].

Given the paucity of data, pediatric CRC treatment protocols are largely extrapolated from adult regimens. Standard treatment includes surgical tumor resection with lymph node clearance. For advanced or high-risk localized disease, adjuvant chemotherapy, typically fluorouracil-based regimens, is recommended, though its efficacy in children remains unclear [12, 13].

Our case underscores the need for heightened clinical suspicion and early intervention in pediatric CRC, especially in resource-limited settings. Delayed diagnosis remains a critical issue. For instance, a similar case in India described a pediatric CRC patient misdiagnosed and treated for abdominal tuberculosis, a presentation echoed in our context [8]. Persistent or atypical symptoms should prompt further investigation to avoid delays in diagnosis.

To improve outcomes, research must prioritize strategies for early detection, especially in low-resource settings. Screening programs tailored to children with persistent gastrointestinal symptoms could enhance early diagnosis and management. Additionally, expanding access to genetic testing and multidisciplinary care in underserved regions is critical.
